# Resistance Training Is an Effective Tool against Metabolic and Frailty Syndromes

**DOI:** 10.4061/2011/984683

**Published:** 2010-12-13

**Authors:** Jan Sundell

**Affiliations:** Department of Medicine, University of Turku, P.O. Box 52, 20521 Turku, Finland

## Abstract

Metabolic syndrome is a set of risk factors (abdominal obesity, insulin resistance, hypertension, and dyslipidemia) which increases markedly the risk of arteriosclerotic vascular disease. In subjects with frailty syndrome, aging-related loss of muscle (sarcopenia) and bone (osteoporosis) might progress to the extent that an older person loses his or her ability to live independently. Due to ongoing obesity pandemic and growing elderly population, metabolic and frailty syndromes are major emerging concerns in healthcare system. Recent studies show that resistance training has remarkable beneficial effects on the musculoskeletal system including prevention and treatment of these syndromes. Resistance training has favourable effect on metabolic syndrome since it decreases fat mass including abdominal fat. It also enhances insulin sensitivity, improves glucose tolerance, and reduces blood pressure values. The combination of sarcopenia and osteoporosis is often seen in the frailty syndrome. Resistance training is probably the most effective measure to prevent and treat sarcopenia. In addition, many studies show that resistance training can maintain or even increase bone mineral density. Optimal nutrition enhances the anabolic effect of resistance training. Resistance training should be a central component of public health promotion programs along with an aerobic exercise.

## 1. Introduction

Resistance training is any activity that causes muscles to contract against external force. Since the goal of resistance training is to progressively overload the musculoskeletal system, weight machines, dumbbells, and barbells are usually used as a resistance. Studies demonstrate that regular progressive resistance training develops the strength and size of muscles [1] and increases bone mass [2] from young male athletes to older women. In addition, resistance exercise might be even more beneficial than aerobic exercise for fat loss [3].

Traditionally, public health guidelines primarily focus on aerobic exercise, which mainly enhances cardiorespiratory fitness. Due to ongoing obesity pandemic and growing elderly population, metabolic [4] and frailty syndromes [5] are major emerging concerns in healthcare system. Recent studies show that resistance training has remarkable beneficial effects on the musculoskeletal system including prevention and treatment of these syndromes. The combination of resistance and aerobic training probably yields the greatest benefits in metabolic syndrome. Therefore, resistance training should be also a central component of public health promotion programs. This paper reviews the effect of resistance training on metabolic syndrome, sarcopenia, and osteoporosis. In addition, advices is given for clinicians on how to perform an effective resistance training including nutritional aspects.

## 2. The Effect of Resistance Training onMetabolic and Frailty Syndromes

### 2.1. Metabolic Syndrome

Metabolic syndrome is a set of risk factors that includes abdominal obesity, insulin resistance (a decreased ability to process glucose), hypertension, and dyslipidemia [4]. This combination of medical disorders increases markedly the risk of arteriosclerotic vascular disease. The prevalence of metabolic syndrome is rapidly increasing worldwide, largely as a consequence of the ongoing obesity pandemic. 

#### 2.1.1. Abdominal Obesity and Insulin Resistance

Abdominal obesity and insulin resistance are the key features of the metabolic syndrome. At rest, skeletal muscle consumes 54.4 kJ/kg (13.0 kcal/kg) per day which is larger than adipose tissue at 18.8 kJ/kg (4.5 kcal/kg) [6]. Since resistance training increases muscle mass, it does not result in weight loss without caloric restriction. However, resistance training, even without caloric restriction, has favourable effect on body composition since it decreases fat mass including abdominal fat [7–9]. Treuth et al., in 1995, [10] already studied over a decade ago the effects of resistance training on body composition. They found in 14 older women who exercised 3 times per week for 16 weeks that although there was no significant change in body weight because of increased muscle mass, intraabdominal adipose tissue was significantly reduced.

Skeletal muscle is an important determinant of insulin sensitivity. In many studies, resistance training has enhanced insulin sensitivity and improved glucose tolerance [9]. Meta-analysis of randomised controlled 9 trials evaluated 372 subjects with type 2 diabetes [11]. When compared to not exercising, progressive resistance training led to small but statistically significant absolute reductions of 0.3% in HbA1c indicating that resistance training is a feasible option in the management of glycemic control in diabetic subjects. Bweir et al. in 2009 [12] demonstrated that resistance training lowers HbA1c in up to 18% in subjects with type 2 diabetes. In this recent study, the resistance training group used 7 exercises that encompassed knee and hip flexion/extension, shoulder flexion/extension, adduction/abduction, elbow flexion/extension, and a chest press. 3 sets of 8–10 repetitions were performed for all exercises with a rest period of 2 minutes between sets in 30–35 minutes 3 times per week for 10 weeks.

Improved glucose uptake appears to be not a mere consequence of the increased muscle mass associated with resistance training but seems to be also a result of qualitative changes in resistance-trained muscle. Holten et al. in 2004 [13] studied the effect of resistance training (30 minutes 3 times per week for 6 weeks) on one leg while the other leg remained untrained. They found that resistance training enhances insulin action in skeletal muscle in type 2 diabetic and healthy subjects. This effect was likely independent of the increases in muscle mass. They concluded that the increases in muscle glucose transporter isoform 4 (GLUT4) content and in various insulin signaling protein expressions and/or activity are part of the mechanism behind the improvement in insulin action.

#### 2.1.2. The Other Clinical Components of Metabolic Syndrome

Resistance training increases blood pressure values acutively and is not therefore recommended for patients with labile hypertension. However, a meta-analysis of 9 randomized controlled trials demonstrated a net reduction of diastolic blood pressure of 3.5 mmHg (*P* < .01) and a nonsignificant reduction of systolic blood pressure of 3.2 mmHg associated with resistance training [14]. Finally, the effects of resistance training on blood lipids are inconsistent. Resistance training might have some effect on the low-density lipoprotein cholesterol (LDL-C) levels since almost half of trials showed significant reductions in LDL-C levels ranging from 5% to 23% [15].

### 2.2. Frailty Syndrome

The frailty syndrome is a collection of symptoms or markers mainly due to the aging-related loss and dysfunction of skeletal muscle and bone. Subjects with the frailty syndrome have increased risk of adverse events such as death, disability, and institutionalization [5]. The costs of frailty syndrome will increase dramatically as the elderly population grows over the next decade.

#### 2.2.1. Sarcopenia

Sarcopenia is the age-related loss of muscle mass and strength. It reflects a progressive withdrawal of anabolism and an increased catabolism, along with a reduced muscle regeneration capacity. Sarcopenia is an important independent predictor of disability linked to poor balance, gait speed, falls, and fractures. Sarcopenia is mediated by multiple mechanisms including sedentary lifestyle, malnutrition, alpha-motor neuron death, altered hormone concentrations and increased inflammation [16]. Resistance training is currently the most effective known strategy to combat sarcopenia by stimulating hypertrophy and increasing strength [17]. 

Typically, the strength increments associated with resistance training have been much larger than the hypertrophic response. Strasser et al. in 2009 [18] demonstrated that after 6 months of resistance training for 3 times per week maximum, strength was increased by an average of 15% for leg press (*P* < .01), 25% for bench press (*P* < .01), 30% for bench pull (*P* < .001), and lean body mass was increased by 1.0 ± 0.5 kg in elderly adults. Based on this randomized controlled trial, they concluded that loading intensity to promote hypertrophy with resistance training should approach 60–80% of one repetition maximum (1RM) with an exercise volume ranging from 3 to 6 sets per muscle group per week of 10–15 repetitions per exercise. Progressive resistance training is an effective and safe tool against sarcopenia also in the very old geriatric patients. Binder et al. in 2005 [19] studied the effects of resistance training on 91 in community-dwelling subjects with frailty syndrome (78 years and older) in a randomized, controlled trial. 3 months of supervised progressive resistance training induced improvements in maximal voluntary thigh muscle strength and whole body fat-free mass in these elderly women and men. In general, significant ameliorations (up to >50% strength gain) can be expected even after 6 weeks of resistance training at a rhythm of 2-3 sessions per week. Therefore, from a preventive viewpoint, all elderly subjects should be advised to start such an exercise program and continue it as long as possible [20].

#### 2.2.2. Osteoporosis

Osteoporosis is a condition resulting in an increased risk of skeletal fractures due to a reduction in the density of bone tissue. The underlying mechanism in all cases of osteoporosis is an imbalance between bone resorption and bone formation [21]. Osteoporosis can be thought of as a bone analog of sarcopenia. Bone remodeling occurs in response to physical stress. Conversely, physical inactivity can lead to significant bone loss. However, not all exercise modalities have shown positive effects on bone mass. For example, unloaded exercise such as swimming has no impact on bone mass, while walking or running has limited positive effects [22]. Many studies have shown that resistance training can maintain or even increase bone mineral density in postmenopausal women [23]. It seems that a combination of high-impact (i.e., jumping) and weight-lifting exercises might be superior for bone stimulation in adults [22]. 

Chilibeck et al. in 2005 [24] studied the effect of resistance training on bone mineral density in 29 older men (age 71 years). Bone mineral density was determined by dual energy X-ray absorptiometry before and after training. They found that resistance training, already after 12 weeks, significantly increased whole-body and leg bone mineral density by approximately 0.5%–1%. The BEST (Bone-Estrogen Strength Training) [25] project identified six specific resistance training exercises that yielded the largest improvements in bone mineral density. This project suggested squat, military press, lateral pulldown, leg press, back extension, and seated row, with 3 weight training sessions a week of 2 sets of each exercise, alternating between moderate (6–8 reps at 70% of 1RM) and heavy (4–6 reps at 80% of 1RM).

## 3. How to Perform an Effective ResistanceTraining

Resistance training and adequate nutrition are needed for muscle and bone growth. In addition, appropriate rest period is required to recover from each exercise. Resistance training impacts several body systems including muscular, skeletal, metabolic, neural, respiratory, and endocrine systems. An understanding and appreciation of basic scientific principles related to resistance training is necessary in order to optimize training responses.

### 3.1. General Aspects

Resistance training is a safe form of exercise when the movements are slow, controlled, and carefully defined also in elderly people [1]. It works by causing microscopic damage to the muscle cells (catabolism), which in turn are quickly repaired (anabolism). The muscle cells adapt to the extra workload by enlarging (hypertrophy) and recruiting greater numbers of nerve cells to aid contraction [26, 27]. One of the fundamentals of effective resistance training is to perform each set or at least the last set(s) of an exercise to fatigue, the state where the subject cannot lift one more repetition with good form [28]. Higher training efficiency is superior to lower one in improving muscle strength [29] and bone mineral density [2]. Another fundamental is the principle of progressive overload in which the workload is gradually increased over time [30]. 

1RM is the maximum amount of weight one can lift in a single repetition for a given exercise. To increase specially muscle size (but also clearly strength), medium to heavy loading is needed (70–80% of 1RM). In general, 1 to 5 repetitions per set performed to fatigue develop especially muscle strength, 8 to 12 reps muscle size, and more repetition muscle endurance. When the training goal is muscular hypertrophy, sets with short rest intervals (about 1 minute) might be superior whereas longer resting periods (several minutes) increase absolute strength [31]. Optimal exercise time is below 60 minutes since thereafter training intensity reduces significantly. Resistance training under the supervision of a health care professional or personal trainer leads to greater workout intensities and is therefore more efficient [32]. To receive lasting results, resistance training should be uninterrupted and be a part of lifestyle. If lack of time is a barrier, 1 training session per week might be enough to maintain the gained results.

### 3.2. Resistance Training Programs in Studies of Metabolic and Frailty Syndromes

In clinical studies of metabolic syndrome, sarcopenia, and osteoporosis, the exercise program provides a total body resistance training program for all of the major muscle groups. Typically, there are 3 training sessions per week, but 2 times might be sufficient too [33]. Before each session, warm up exercises are performed for about 5–10 minutes, consisting of stretching exercises for the major muscle groups. Thereafter, 5–15 exercises are performed including leg press, squat, leg extension, leg curl, heel raise, bench press, shoulder press, lateral pulldown, seated row, back extension, sit-up, tricep extension, and arm curl. Large muscle groups are worked before smaller ones. Usually 1–4 sets of 8–15 repetitions (at 50–85% of 1RM) often performed to fatigue have been used for all exercises with a rest period of 0.5–2 minutes between sets (Table 1). The regular time required to complete the resistance training program is within 45 minutes, in many studies half an hour.

### 3.3. Resistance Training Programs in Future Studies

In previous studies, each large muscle group has been usually trained for 3 times per week. However, more advanced practitioners like bodybuilders and experts in muscle growth split the training program [34]. Split training involves working no more than two or three muscle groups or body parts per day. It involves fully exhausting individual muscle groups during a workout, then allowing several days for the muscle to fully recover. Muscles are trained once or twice per week and allowed roughly 72 hours to recover (Table 2). In future studies of metabolic and frailty syndromes, it would be important to compare the effect of whole-body and split resistance training programs. In addition, resistance training periods have been quite short in previous studies (up to 6 months). Since less is known about long-term hypertrophic response to resistance training in older adults, studies with years of training are needed. Finally, varying the number of repetitions and length of rest and to some extent the types of exercises over time (periodization), it may be possible to achieve greater benefits and hypothetically decrease the risk of injury.

## 4. Nutrition

Optimal nutrition enhances the anabolic effect of resistance training. It seems to be reasonable to eat at regular intervals and split daily food intake into 4 to 6 protein-rich meals instead of traditional 3 meals a day.

### 4.1. Meals and Protein Supply

Protein is needed for cell growth and repair. In adults, daily protein supply, measured as intake per body weight, is 0.8–1.0 g/kg. While protein restriction may be appropriate for treatment of existing kidney disease, there is no significant evidence for a detrimental effect of high-protein intakes on kidney function in healthy subjects [35]. Several studies have concluded that active people and athletes require elevated protein intake [36]. The anabolic effect of resistance exercise is enhanced by the provision of dietary protein. In intensive resistance training, adequate protein supply, from 1.5 to 2 g/kg per day [37], is required for maximal muscle growth. Daily protein supply should be divided constantly on several meals (~20 g per meal) [38]. Also an adequate supply of carbohydrates is needed as a source of energy and for the body to restore glycogen levels in muscles. Light, balanced meal prior to the workout ensures that adequate energy and amino acids are available for the intense bout of exercise. Water should be consumed throughout the course of the workout to prevent poor performance due to dehydration.

### 4.2. Nutritional Supplements

Nutritional supplements are very popular especially among athletes although some studies show either controversial or even negative results. However, whey protein and creatine seem to have significant positive effects on muscle size, strength, and athletic performance without major adverse effects and high costs. Most studies have shown that supplementation of whey (~15–30 g) alone or with carbohydrates immediately after and possibly before and during resistance exercise can enhance the muscle hypertrophy response to resistance training [39]. Some studies also suggest that whey protein may enhance recovery from heavy exercise and possibly decrease muscle damage and soreness [37]. 

Creatine supplementation increases the intracellular pool of phosphocreatine. Phosphocreatine provides a reserve of energy to rapidly regenerate ATP, which is consumed as a result of muscle contraction. The most common creatine loading programme involves an initial loading phase of 20 g/day for 5–7 days, followed by a maintenance phase of 3–5 g/day for differing periods of time (1 week to 6 months). Carbohydrate ingestion augments creatine retention during creatine feeding [40]. Creatine has been studied in hundreds of clinical trials and has shown benefits including increased muscle strength, power, and size [41] also in elderly men and women [42, 43]. In addition, creatine might increase bone mineral content may be due to an enhanced muscle mass, with potentially greater tension on bone at sites of muscle attachment [24]. Creatine supplementation causes minor water retention (1-2 kg) but has no severe adverse effects such as renal dysfunction [44]. However, creatine supplementation should not be used by an individual with existing renal disease, and more studies to address the issue of long-term (for several years) creatine usage are needed.

### 4.3. Nutritional Options in Future Studies

The effect of nutrition on metabolic and frailty syndromes during resistance training has not been investigated accurately. In especially frailty syndrome, protein malnutrition is common and nutritional intake might be insufficient to maintain adequate total body mass in these subjects. Esmarck et al. in 2001 [45] demonstrated that after resistance training immediate intake of a protein supplement is more effective than delayed intake for the maximal development of muscle hypertrophy in elderly men. It is possible that optimal nutrition would improve the results in clinical studies. Therefore, in future studies, nutritional evaluation is indicated including adequate protein supply (e.g., 1.5 g/kg per day) and a whey protein shake immediately following the exercise, because both protein uptake and protein usage are increased at this time (Table 3). Finally, some subjects, for example, in frailty syndrome, might benefit from creatine supplementation. Creatine is very inexpensive, 1 kg costs about 30 euros and is almost enough for a year of usage.

## 5. Conclusion

The beneficial effects of resistance training have been known for decades in physiatrics and rehabilitation medicine. However, recent studies demonstrate major positive actions far beyond this. The prevalence and costs of metabolic and frailty syndromes will increase dramatically due to ongoing obesity pandemic and growing elderly population. Resistance training is an effective tool against these syndromes. Resistance training has favourable effect on body composition since it decreases fat mass including abdominal fat. It also enhances insulin sensitivity, improves glucose tolerance, and reduces blood pressure values. The combination of sarcopenia and osteoporosis results in the significant frailty syndrome often seen in the elderly population. Sarcopenia may progress to the extent that an older person may lose his or her ability to live independently. Resistance training is probably the most effective measure to prevent and treat sarcopenia. In addition, many studies have shown that resistance training can maintain or even increase bone mineral density. Therefore, resistance training should be a central component of public health promotion programs along with an aerobic exercise. More studies are needed to identify the effect of different training programs, long-term responses, and the role of optimal nutrition to enhance the anabolic effects of resistance training. It is time for muscle millennium.

## Figures and Tables

**Table 1:** An example program which mimics different resistance training protocols used in clinical studies of metabolic syndrome, sarcopenia, and osteoporosis.

Exercise	Sets	Repetitions
Leg press	3	8–12
Leg extension	3	8–12
Leg curl	3	8–12
Lateral pulldown	3	8–12
Bench press	3	8–12
Shoulder press	3	8–12
Back extension	3	8–12

3 resistance training sessions per week (nonconsecutive days). Before each session, warm up exercises are performed for about 5–10 minutes. At least the last set should be performed to fatigue. A rest period between sets is 1-2 minutes and typical time required to complete the program is within 45 minutes.

**Table 2:** An example of a partially split resistance training program.

Day	Muscle	Exercise	Sets × Repetitions
(I) Lower body, for example, Monday	Musculus gluteus and femur	Hack squat	3 × 8–12
	Musculus gluteus and femur	Leg press	3 × 8–12
	Musculus quadriceps femoris	Leg extension	3 × 8–12
	Musculus biceps femoris	Leg curl	3 × 8–12
	Musculus gastrocnemius	Standing calf raise	3 × 8–12
	Musculus erector spinae	Back extension	3 × 8–12
	Musculus rectus abdominis	Sit up	3 × 8–12

(II) Upper body, for example, Tuesday	Musculus latissimus dorsi	Lateral pulldown	3 × 8–12
	Musculus pectoralis	Bench press	3 × 8–12
	Musculus deltoideus	Shoulder press	3 × 8–12
	Musculus triceps brachii	Triceps pressdown	3 × 8–12
	Musculus biceps brachii	Biceps curl	3 × 8–12
	Musculus erector spinae	Back extension	3 × 8–12
	Musculus rectus abdominis	Sit up	3 × 8–12

(III) Whole body, for example, Friday	Musculus gluteus and femur	Leg press	3 × 8–12
	Musculus quadriceps femoris	Leg extension	3 × 8–12
	Musculus biceps femoris	Leg curl	3 × 8–12
	Musculus gastrocnemius	Standing calf raise	3 × 8–12
	Musculus latissimus dorsi	Lateral pulldown	3 × 8–12
	Musculus pectoralis	Bench press	3 × 8–12
	Musculus deltoideus	Shoulder press	3 × 8–12

In the present program each large muscle group is trained 2 times per week.

**Table 3:** Proposal for protein intake during resistance training program in future clinical studies.

Meal	Protein supply	Source
Breakfast	20 g	Protein supplement, for example, whey protein shake, if necessary
Lunch	20 g	Fish, chicken or low-fat meat
Snack	20 g	Fat-free cottage cheese or curd
Recovery drink, if exercising daily	20 g	Whey protein shake
Evening meal	20 g	Fat-free cottage cheese or curd

This proposal assumes that the weight of the subject is 70 kg.

## Abbreviations

LDL-C: Low-density lipoprotein cholesterol
1RM: One repetition maximum.
